# Retraction Note: Violence and depression among pregnant women in Egypt

**DOI:** 10.1186/s12884-025-08276-7

**Published:** 2025-10-11

**Authors:** Hanan M. Ghoneim, Mohamed Elprince, Tamer Yehia M. Ali, Waleed F. Gharieb, Amal A. Ahmed

**Affiliations:** Department of Obstetrics and Gynecology, Faculty of Medicine, Suez Canal University, Round Road, Ismailia, Egypt

**Retraction Note: BMC Pregnancy and Childbirth 21, 502 (2021)**

**https://doi.org/10.1186/s12884-021-03932-0**

The Editor has retracted this article because the authors did not provide evidence that ethics approval had been received before the commencement of this study. Hanan M. Ghoneim, Mohamed Elprince, and Waleed F. Gharieb disagree to this retraction. Tamer Yehia M. Ali and Amal A. Ahmed did not respond to correspondence from the Editor about this retraction.

